# Yes, research matters

**DOI:** 10.1371/journal.ppat.1006420

**Published:** 2017-08-10

**Authors:** Mari L. Shinohara

**Affiliations:** 1 Department of Immunology, Duke University School of Medicine, Durham, North Carolina, United States of America; 2 Department of Molecular Genetics and Microbiology, Duke University School of Medicine, Durham, North Carolina, United States of America; The Fox Chase Cancer Center, UNITED STATES

## Abstract

My father was diagnosed with stomach cancer recently. Luckily, it was still at an early stage, and endoscopic surgery successfully took care of it. My father was fortunate; since people with stomach cancer do not show clear symptoms in the early stages, the disease is often not diagnosed until it becomes advanced. In his case, the diagnosis started from a suggestion by his doctor to check whether he had a gastric infection with *Helicobacter pylori*, a bacterial species found in the digestive tract. In Japan, where he lives, a majority of gastric cancer patients (more than 99%) have been infected with *H*. *pylori* [1], and the causative role of this bacterial species in promoting gastric cancer is very well established. Now, scientific understanding connecting gastric cancer to *H*. *pylori* is saving the lives of many people, including my father. Thinking about this recent personal experience, I wonder if the connection between bacteria and cancer might have been considered a crazy idea decades ago.

Research makes it possible to connect seemingly unrelated matters. My laboratory works on seemingly unrelated research topics, such as fungal infections and autoimmunity. However, my question is the same whatever the topic: How do leukocytes elicit and regulate inflammation when they detect infections or endogenous signals? In fact, host receptors detecting pathogens can induce autoimmunity, and autoimmunity alters host sensitivity to pathogens due to the imbalance in the immune system.

We are beginning to gain some insight into this question, as revealed by some of our recent studies. For example, the NLR family, pyrin domain containing 3 (NLRP3) inflammasome, which is known to sense a wide variety of pathogens, can also change the course of experimental autoimmune encephalomyelitis (EAE), an animal model of multiple sclerosis (MS). In particular, our study suggested that disease treatment approaches need to be changed based on the activation status of the NLRP3 inflammasome [2]. Another recent study from our laboratory demonstrated that a protein, termed osteopontin (OPN), skews the balance of population sizes between myeloid cells (i.e., innate immunity) and lymphoid cells (i.e., adaptive immunity) during infections and other biological insults [3]. An intracellular isoform of OPN (iOPN) negatively regulates emergency myelopoiesis. Thus, OPN attenuates host resistance by limiting neutrophil supply at the early stage of systemic *Candida* infection. In contrast, a secreted OPN (sOPN) isoform positively regulates the expansion of T lymphocytes and ends up triggering autoimmune colitis.

I am an immunologist but obtained my PhD in mycology. Nevertheless, it took some time for me to appreciate that research enables us to connect the dots placed far apart. This is a truly exciting time to connect seemingly unrelated biological phenomena, because scientists are exponentially increasing our understanding of nature. This is particularly true in innate immunity, which is not only the central alarming system in host–microbe interactions but also relates to almost any human disease we can imagine. However, we are facing a dark age for science and research, in which certain interests wrongfully discredit some research fields. There are things that can be achieved only by research. I am always ready to tell anyone, “Yes, research matters!”.

**Image 1:**
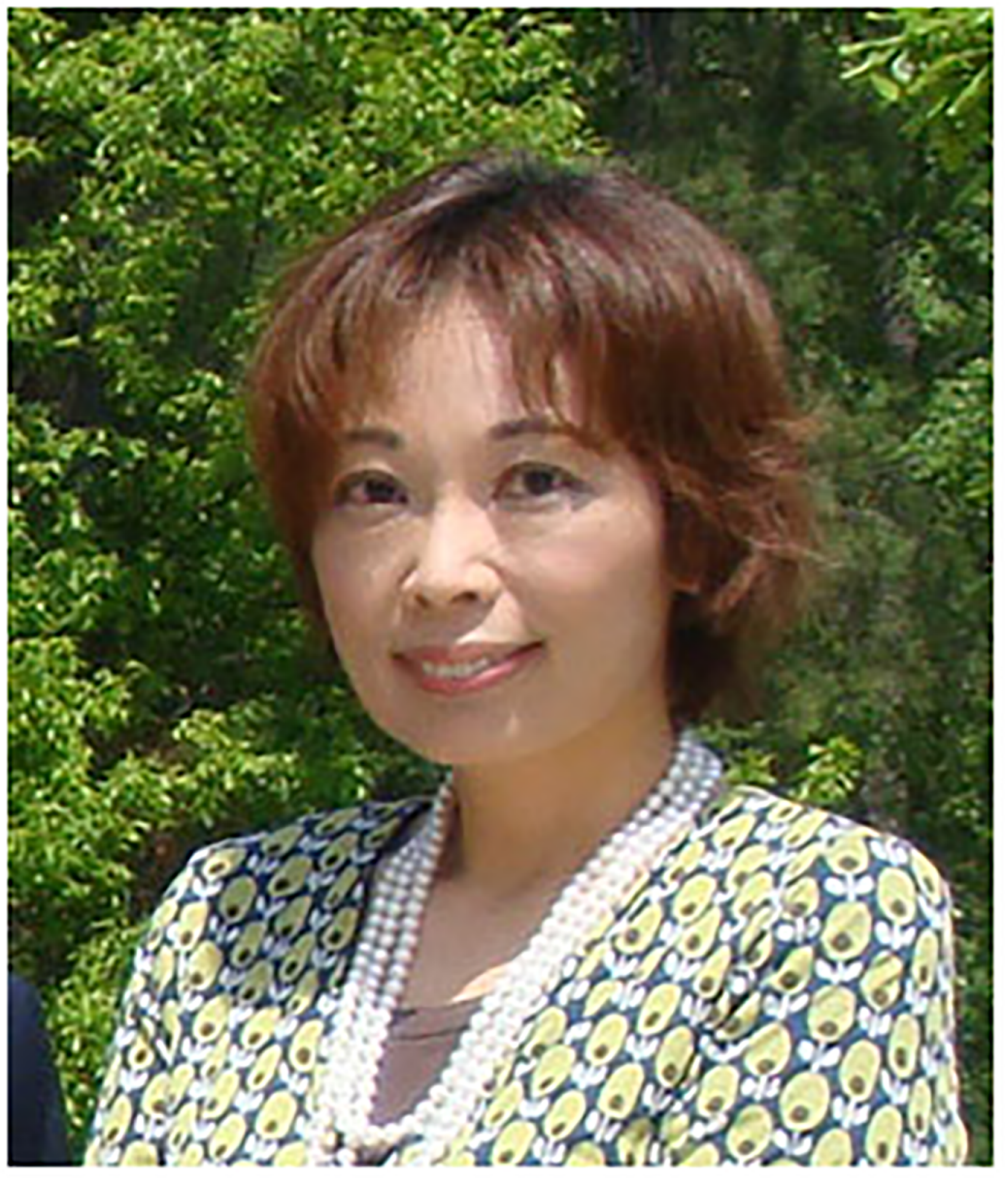
Mari L. Shinohara.
